# Comparative Performance Evaluation Between a Modified Hybrid Dryer and a Commercially-Manufactured Fluidized Bed Agglomerator for Producing Instant Coconut Milk Powder

**DOI:** 10.3390/foods15020210

**Published:** 2026-01-07

**Authors:** Titaporn Tumpanuvatr, Weerachet Jittanit

**Affiliations:** 1 Department of Food Processing and Preservation, Institute of Food Research and Product Development, Kasetsart University, Chatuchak, Bangkok 10900, Thailand; ifrtot@ku.ac.th; 2 Department of Food Science and Technology, Faculty of Agro-Industry, Kasetsart University, Chatuchak, Bangkok 10900, Thailand

**Keywords:** coconut milk powder, agglomerator comparison, fluidized bed agglomerator, glass transition, physical property

## Abstract

This work investigated the comparative performance of two fluidized bed agglomeration systems for producing instant coconut milk powder: a commercially manufactured unit and a hybrid dryer previously modified into a fluidized bed agglomerator. Three binder solutions, distilled water, xanthan gum, and xyloglucan polysaccharide, were employed to examine how equipment configuration and binder type influence key powder properties. The aim was to evaluate the effects of fluidized bed agglomerator design and binder selection on coconut milk powder characteristics, including moisture content, bulk density, solubility, and glass transition temperature. All samples, including the non-agglomerated control, exhibited moisture contents ranging from 2.1% and 2.6% (w.b.), meeting the criterion for safe long-term storage. Powders produced with hydrocolloid binders (xanthan gum and xyloglucan) possessed lower bulk densities than those agglomerated with water, reflecting the formation of more open particle structures. When identical binders were applied, the two agglomerators produced comparable solubility outcomes, although water-based agglomerates consistently dissolved the fastest. Differential scanning calorimetry indicated a substantial increase in glass transition temperature after agglomeration, confirming improved structural stability. Overall, the results demonstrate that both agglomeration systems effectively enhanced the physicochemical and functional characteristics of coconut milk powder, with only minor variations that were attributable to equipment design.

## 1. Introduction

The demand for plant-based milk alternatives, such as soy, almond, oat, chickpea, and coconut milk, has grown rapidly in recent years as consumers seek products aligned with health, environmental, and ethical considerations [1,2]. This market expansion has also stimulated the development of secondary or derivative products derived from these plant-based milks, reflecting a broader diversification of the dairy alternatives sector [3]. Compared with conventional bovine milk, plant-based milks generally provide lower amounts of saturated fat and cholesterol and may contain beneficial levels of dietary fiber and bioactive compounds [4]. However, their intrinsic limitations, such as low protein concentration, unbalanced amino acid profiles, limited micronutrient content, and physical instability during processing and storage, remain significant challenges for product formulation and functional optimization [5].

Coconut milk, produced by extracting the aqueous emulsion from grated kernels of mature coconuts, has become a prominent plant-based ingredient owing to its distinctive aroma, creamy texture, and nutritional profile enriched with medium-chain triglycerides [2]. It is widely employed in culinary formulations, ready-to-drink beverages, and functional food products as a substitute for dairy-based components. However, the high water activity and fat content of fresh coconut milk render it extremely prone to microbial and physicochemical deterioration, restricting its distribution and storage stability [6]. Transforming coconut milk into a dry powder form offers a practical approach to enhance its preservation, reduce transportation costs, and increase versatility for industrial use in instant drinks, desserts, bakery fillings, and convenience foods [4,5]. Among various dehydration technologies, spray drying and drum drying are the most established industrial methods for converting coconut milk into powder while maintaining acceptable quality and reconstitution performance.

Although converting coconut milk into powder offers clear advantages for storage and transport, the resulting spray-dried product typically suffers from limited instant properties such as poor wettability, low dispersibility, and flowability [1,7]. These drawbacks are mainly associated with the fine particle size, surface stickiness, and cohesive characteristics of high-fat powders, which complicate their reconstitution and industrial handling. To overcome these challenges, agglomeration has been widely adopted as a post-drying modification technique to enhance powder functionality. Agglomeration process has shown strong potential for improving the usability of coconut milk powder by producing larger, more porous granules that rehydrate rapidly and exhibit improved flow performance [4,5]. Through controlled particle enlargement and restructuring, agglomeration significantly improves wettability, dispersibility, and flowability, thereby addressing critical functional limitations [4,7].

Fluidized bed agglomeration has been widely applied to improve the instant properties of food powders, particularly wettability, solubility, and reconstitution behavior, through modifications of particle size, structure, and surface characteristics. Various binders, such as maltodextrin and gum Arabic, have been successfully used to enhance agglomeration performance [8,9]. For fat-rich powders, existing published research has mainly emphasized particle stickiness and glass transition behavior, rather than systematic evaluations of different agglomeration systems [10]. In the case of coconut milk powder, which is characterized by its high fat content and thermal sensitivity, available studies have largely concentrated on spray drying and formulation strategies, while investigations on fluidized bed agglomeration and instantization performance remain limited. The presence of lipids and low-molecular-weight carbohydrates further increases particle stickiness, making agglomeration behavior strongly dependent on equipment design, airflow characteristics, and binder application. The instantaneity of powdered products, defined as their ability to dissolve quickly and easily, is closely related to key powder properties, including low moisture content, appropriate bulk density, high solubility, and glass transition temperature. Maintaining these properties during handling, transport, and storage requires careful control of moisture and temperature, the use of moisture-proof packaging, and prevention of particle compaction. Despite the industrial importance of instant coconut milk powder, studies directly comparing the effects of agglomeration systems and binder types on moisture content, bulk density, solubility, and glass transition temperature parameters that govern instantaneity and powder stability are still scarce.

A range of agglomeration strategies, such as integrated agglomeration during spray drying, rewetting-based techniques, and fluidized bed agglomeration, has been explored to enhance the functionality and handling characteristics of food powders [1,11]. Among these methods, fluidized bed agglomeration stands out for its effective heat and mass transfer, better control over particle growth, and capacity to generate agglomerates with uniform size and open, porous structures [5,7]. These features are especially advantageous when processing powders containing heat-sensitive or fat-rich components, such as coconut milk powder. In a typical top-spray fluidized bed agglomeration system, fluidizing air suspends the powder bed while a binder solution is atomized from an overhead nozzle. The sprayed droplets act as temporary adhesive bridges between the suspended particles, initiating agglomerate formation. As the process continues, warm air evaporates the liquid phase, progressively solidifying the interparticle bonds and yielding stable, enlarged granules [12,13]. Through appropriate selection of processing parameters and binder formulations, the fluidized bed agglomeration technique enables targeted modification of particle morphology, porosity, and flow characteristics. The final product quality depends on a combination of material attributes, binder rheology, and operational conditions such as airflow velocity, spray rate, and atomization pressure [14,15,16,17].

The performance and efficiency of the fluidized bed agglomeration process are governed by several interrelated operating variables, including the binder feed rate, atomization height, drying air temperature, and airflow velocity, as well as the physicochemical characteristics of the binder employed [1,11,18]. The choice of binder plays a decisive role in determining particle growth dynamics and the final attributes of the agglomerates. Previous studies have demonstrated that formulations containing maltodextrin, whey protein concentrate, sodium caseinate, or polyvinylpyrrolidone can markedly influence the internal structure, cohesiveness, wettability, and overall reconstitution performance of food powders [4,7]. Achieving powders with desirable porosity, flow properties, and rapid solubility therefore requires careful coordination between binder formulation and process settings to ensure optimal liquid bridging, drying kinetics, and particle consolidation [1,5].

Despite the growing research on fluidized bed agglomeration, comparative analyses of different agglomerator configurations and binder systems remain scarce, particularly for fat-rich, plant-based matrices such as coconut milk powder. In the present investigation, two types of fluidized bed agglomeration equipment were evaluated: a commercially manufactured laboratory-scale unit and a custom-built system previously adapted from a hybrid drying apparatus developed by the authors [19]. The key objective was to evaluate the effects of fluidized bed agglomerator design and the influence of binder type on coconut milk powder characteristics, including moisture content, bulk density, solubility, and glass transition temperature. Three binder solutions, distilled water, xanthan gum, and xyloglucan polysaccharide, were selected to represent liquids with contrasting viscosities and molecular structures, enabling systematic assessment of their effects on particle formation, strength, and instant properties. Moreover, the in-house fabricated agglomerator was benchmarked against the commercially manufactured system to validate its performance. Comparable results between the two units would help confirming that the modified design can reliably replicate commercial-scale operation under laboratory conditions. Overall, this study extends the authors’ prior findings by providing new evidence on how binder rheology and agglomerator configuration jointly determine the functionality of instant coconut milk powder. The insights gained are expected to support future process optimization and pilot-scale applications for producing high-quality, reconstitutable powders in the food industry.

## 2. Materials and Methods

### 2.1. Raw Materials

“Prung” brand commercial coconut milk powder used in the fluidized bed agglomeration trials was supplied by One Thai Foods Co., Ltd. (Bangkok, Thailand). Based on the manufacturer’s product specification, the formulation of the powder included approximately 73.25% fresh coconut milk, 24.75% glucose syrup, and 0.50% sodium caseinate, along with an acidity regulator (INS 340(ii)), an emulsifier (INS 471), and an anticaking agent (INS 551). Xanthan gum was procured from Chemipan Co., Ltd. (Bangkok, Thailand), whereas xyloglucan was supplied by Jittanich Padrew Co., Ltd. (Chachoengsao, Thailand).

### 2.2. Coconut Milk Powder Agglomeration Applying a Commercially Manufactured Agglomerator

Agglomeration experiments were conducted using a commercially manufactured fluidized bed system (Mini-Glatt, Model 19484 B203000; Glatt Ingenieurtechnik GmbH, Weimar, Germany). For each experiment, 100 g of commercial coconut milk powder was loaded into the chamber as the initial sample. Three binder formulations, distilled water, 1% *w*/*w* xanthan gum solution, and 1% *w*/*w* xyloglucan solution, were employed. All binder solutions were prepared at ambient temperature prior to the experiments. To ensure complete solubilization, the xyloglucan dispersion was left to hydrate overnight before agitation, whereas the xanthan gum solution was freshly mixed on the day of use due to its rapid dissolution characteristics. A two-fluid nozzle with a 0.8 mm orifice was utilized to atomize the binder, with compressed air maintained at 0.2 MPa. The manufacturer’s configuration positioned the nozzle 12 cm above the air flow distributor plate, with a diameter of 7.5 cm. The inlet air temperature was kept constant at 50 °C throughout all trials. The relative humidity of the inlet air was not controlled; however, due to heating of the ambient air to 50 °C, the relative humidity was expected to decrease and remain below approximately 30%. After contacting the powder bed, the drying air was discharged directly to the surroundings without recirculation. The binder feed rate varied among treatments, as summarized in Table 1. Binder application followed an intermittent cycle consisting of one minute of spraying followed by a one-minute pause, promoting uniform wetting and gradual agglomerate formation. Once sufficient granule development was visually observed, drying continued for an additional 50–60 min to stabilize the agglomerates. The total binder volume delivered to each batch is detailed in Table 1.

During processing, the air flow rate was carefully adjusted to maintain a stable fluidized state while preventing powder flowing out of the drying chamber. Air flow was typically controlled within 8–28 m^3^/h, corresponding to an estimated air velocity of 0.5–1.8 m/s. Initially, the air flow was set to a lower level to accommodate the fine native powder and progressively increased as particle size and mass grew due to binder deposition and agglomerate consolidation. Because wetting, drying, and particle circulation occur simultaneously in the fluidized bed agglomeration process, fine-tuning of these parameters was necessary to sustain uniform fluidization and consistent agglomerate growth. The selected experimental settings were refined through a series of preliminary tests performed with the same commercially manufactured apparatus.

### 2.3. Coconut Milk Powder Agglomeration Using the Modified Fluidized Bed System

The second agglomeration system employed in this study was a laboratory-scale unit custom-designed and fabricated by the authors. This apparatus originated from the hybrid dryer previously developed by Tumpanuvatr et al. [20] and later adapted for agglomeration applications [19]. The same unit was utilized here to further optimize the process and to benchmark its performance against the commercially manufactured fluidized bed system. In brief, the hybrid dryer integrated a fluidized bed and a heat pump drying mechanism with a single cylindrical drying chamber (30 cm in diameter and 100 cm in height) operating in batch mode. The fluidized bed section was equipped with two 2.2 kW high-pressure blowers (B.O. Intelligence Co., Ltd., Bangkok, Thailand) whose speeds were regulated by inverters, together with a 36 kW electrical heater to provide thermal energy. The drying temperature was controlled by a proportional–integral–derivative (PID) controller with ±1 °C precision. The heat pump subsystem was composed of a 2.2 kW hermetic scroll compressor (Copeland, Emerson Climate Co., Ltd., St. Louis, MO, USA), an evaporator powered by a 0.158 kW centrifugal fan (model DDV 25, Uniaire Corporation Co., Ltd., Samutprakarn, Thailand), and dual air-cooled condensers (internal and external; model ACV 25, Uniaire Corporation Co., Ltd., Samutprakarn, Thailand). Both subsystems shared the same drying chamber and distributor configuration, and the refrigerant used in the heat-pump circuit was R-22. A supplementary 0.746 kW blower (B.O. Intelligence Co., Ltd., Bangkok, Thailand) circulated the air through the heat-pump loop. Full details of the dryer’s structure and operation are available in Tumpanuvatr et al. [20].

To transform the hybrid dryer for operation as a fluidized bed agglomerator, a binder-spraying module was incorporated to enable simultaneous spraying and fluidization. Comprehensive details regarding the modification and operational principles of the system are provided by Tumpanuvatr et al. [19] The additional components consisted of a Vergin oil-free air compressor (model WP550-1-30L, Dragon Rich Co., Ltd., Nonthaburi, Thailand) upgraded from a 0.55 kW to a 1.0 kW motor. The system was equipped with a pressure regulator to stabilize the inlet air pressure and maintain a consistent airflow during operation. A Seko-brand peristaltic pump (model PPR0301A2000-A, Seko Fluid Controls (Thailand) Co., Ltd., Bangkok, Thailand), and an Ikeuchi-brand full-cone pneumatic nozzle (model BIMJ-7004-S303, Siam Ikeuchi Co., Ltd., Bangkok, Thailand) with a 0.4 mm orifice, together with the necessary tubing and fittings.

During agglomeration, only the fluidized bed section of the system was operated; the heat-pump circuit remained inactive. The binder spray was directed vertically downward toward the powder bed, with the nozzle positioned 7 cm above the distributor plate inside the chamber. The distributor was manufactured from stainless steel and included 2 mm perforations with hooded openings set at a 5 mm pitch, an arrangement previously identified as producing the greatest air velocity and swirling pattern in the bed in comparison with other types [20].

At the start of each run, one blower was operated at 840 rpm (approximately 1.5 m/s air velocity) to prevent the fine particles from being carried away from the chamber. After 5 min, the rotation speed was raised to 2800 rpm (≈3.0 m/s) and maintained at that level throughout the remainder of the process. This staged increase was necessary because, as agglomeration progressed and particle mass and size increased, higher air velocities were required to sustain proper fluidization.

All other experimental variables, including sample mass, binder formulations and preparation, air pressure at the nozzle, drying temperature, and intermittent spray–pause cycles, were identical to those described for the Mini-Glatt system. The specific binder feed rates, total binder volumes, and drying durations used for each experimental condition are presented in Table 1. Similarly to the commercially manufactured agglomerator, the relative humidity of the inlet air was not controlled, and the drying air was exhausted to the surroundings after contacting the powder bed without recirculation. These operating parameters were refined based on preliminary trials to ensure reproducible agglomerate formation with the modified system. A summary comparing the principal design and operating characteristics of the commercially manufactured and modified agglomerators is provided in Table 2.

### 2.4. Quality Determination

The physical and functional properties of the agglomerated coconut milk powder samples were evaluated and compared with those of the original (non-agglomerated) powder. Parameters assessed included moisture content, tapped bulk density, solubility, and glass transition temperature. Each measurement was performed in triplicate.

#### 2.4.1. Moisture Content

Moisture determination was carried out using a standard thermogravimetric approach. About 2 g of sample was accurately weighed into a pre-dried aluminum dish and heated in a hot air oven at 105 °C until the sample reached a constant mass. The procedure followed the guidelines of the AOAC [21].

#### 2.4.2. Tapped Bulk Density

Tapped bulk density was measured according to the method of Beristain et al. [22] with minor adjustments. A 50 g portion of each powder sample was transferred into a graduated cylinder and manually tapped 100 times on a resilient rubber surface to achieve proper compaction. The tapped bulk density (g/mL) was then computed as the ratio of the sample mass to the final settled volume within the cylinder.

#### 2.4.3. Solubility

The solubility test was performed following the procedure of Don et al. [23] with slight modification. Approximately 1 g of powder was dissolved in 50 mL of distilled water maintained at ambient temperature (around 25 °C). The mixture was stirred in a glass beaker using a magnetic stirrer (IKA C-MAG HS 7, Rawang, Selangor, Malaysia) operating at speed level 2, equivalent to about 570 rpm. The dissolution process was observed visually, and the time taken for all visible particles to disappear was recorded as the solubility value for each sample.

#### 2.4.4. Glass Transition Temperature

The glass transition temperature is a fundamental thermophysical property that indicates the temperature range at which amorphous solids transition from a rigid glassy phase to a more mobile rubbery state. Although the glass transition temperature is not itself a direct measure of product quality, it provides valuable insight into powder stability and functional behavior, particularly regarding stickiness, flowability, and dissolution performance. Jaya and Das [24] reported that the sticky point of food powders, closely related to the glass transition temperature affects both solubility and handling, as materials above their glass transition temperature tend to soften and aggregate. In this study, the glass transition temperature was determined to evaluate how binder composition and agglomerator configuration influence the structural stability of the resulting powders.

Differential scanning calorimeter (DSC) was used to determine the glass transition temperature. Measurements were performed using a Pyris Diamond DSC (PerkinElmer Inc., Waltham, MA, USA) controlled by Pyris software (PerkinElmer Inc., Waltham, MA, USA). The analytical procedure was based on Hedayatnia et al. [25] with minor modifications. Approximately 8 mg of sample was sealed in a 40 μL hermetic aluminum pan (ME-26763; Mettler-Toledo International Inc., Columbus, OH, USA), with an empty pan used as the reference. Each sample was equilibrated at 25 °C for 1 min, then heated from 25 °C to 150 °C at a rate of 10 °C/min, followed by a 1 min hold at 150 °C under a nitrogen atmosphere.

### 2.5. Statistical Analysis

All experimental data were analyzed following a completely randomized design. Results are presented as mean ± standard deviation from three independent replicates. Analysis of variance (ANOVA) was applied to evaluate differences among treatment means, and post hoc comparisons were performed using Duncan’s multiple range test at a significance level of *p* ≤ 0.05. All statistical procedures were carried out using IBM SPSS Statistics software, version 23 (IBM Corp., Armonk, NY, USA).

## 3. Results and Discussion

Table 3 summarizes the measured values of moisture content, tapped bulk density, solubility, and glass transition temperature for all samples, including those produced using the commercially manufactured and modified fluidized bed agglomerators as well as the original coconut milk powder prior to agglomeration.

The moisture contents of both the unagglomerated coconut milk powder (initial material) and the agglomerated powders produced with the two different systems were consistently low, ranging from 2.1% to 2.6% on a wet basis (w.b.). These values indicate that all powders possess adequate dryness for stable storage under ambient conditions. In accordance with the Codex Alimentarius Commission [26], milk powder products should not exceed 5% moisture (w.b.), and all samples in this study were well below that threshold. Despite differences in equipment configuration and airflow characteristics between the commercially manufactured and modified agglomerators, comparable final moisture levels were obtained through appropriate adjustment of operating parameters. The results obtained were slightly less than the typically values reported for spray-dried coconut milk powder (2.75% and 4.95% w.b.) and probiotic-enriched almond milk powder (2.83–3.26% w.b.) as documented by Fatimah et al. [27] and Lipan et al. [28], respectively. All agglomerated powders exhibited markedly lower tapped bulk density values than the original coconut milk powder. This outcome reflects the effectiveness of the fluidized bed agglomeration process in generating larger, more open granule structures through the adhesion of fine particles into porous clusters. Such a structural rearrangement is expected to improve powder reconstitution behavior, particularly solubility, as discussed in the following section [29]. The relatively high density of the unagglomerated coconut milk powder arises from its compact, closely packed particles, whereas the agglomerated products possessed greater particle volume without a corresponding rise in mass, leading to a lower overall bulk density. The extent of agglomeration and the associated structural changes in coconut milk powder produced using the modified hybrid dryer were previously investigated by the authors [19]. In that study (Tumpanuvatr and Jittanit [19]), particle size distributions of the non-agglomerated coconut milk powder and agglomerated samples prepared using the same three binders as in the present work were determined using a standard dry sieving method. The results showed that the majority of the non-agglomerated coconut milk powder (approximately 70% by weight) consisted of particles in the size range of 0.15–0.25 mm (corresponding to mesh sizes 60–100). In contrast, agglomeration led to a pronounced shift toward larger particle sizes. Specifically, powders agglomerated using distilled water exhibited particle sizes predominantly in the range of 0.25–0.5 mm (approximately 60% by weight), whereas those produced using xanthan gum and xyloglucan solutions showed even greater particle growth, with the dominant fraction falling within the 0.5–1.0 mm range (approximately 60% and 53% by weight, respectively). These previously reported particle size distributions are consistent with the reductions in bulk density observed in the present study.

When comparing the two agglomeration systems, powders produced with the modified hybrid-dryer unit showed noticeably lower tapped bulk density than those obtained from the commercially manufactured fluidized bed agglomerator under identical binder conditions. This difference can be attributed to the distinct operational characteristics between the two machines, such as binder delivery rate, total binder volume, nozzle orifice diameter (which affects droplet size), nozzle height located above the airflow distributor plate, drying chamber arrangement, and drying air velocity. Because these variables interact in a highly interdependent manner, collectively influencing granule growth, porosity development, and drying behavior, their effects on bulk density were interpreted as a combined outcome rather than isolated factors.

The incorporation of hydrocolloid binders, xanthan gum and xyloglucan, produced agglomerates with markedly lower tapped bulk density compared with those formed using distilled water. Ji et al. [15] reported a comparable observation, noting that water, owing to its low viscosity, tends to generate more compact agglomerates due to the closer packing of primary particles. In contrast, the higher viscosity of polysaccharide-based binders promotes the development of larger granules with open, porous structures and reduced packing efficiency, thereby lowering the apparent density. This explanation is in agreement with the findings reported by Dewettinck et al. [30], who examined top-spray fluidized bed coating of glass beads using various hydrocolloids, locust bean gum, high- and low-viscosity carboxymethylcellulose, sodium alginate, and carrageenan, and concluded that the agglomeration behavior was primarily governed by the rheological characteristics of the wet film formed during processing rather than the intrinsic properties of the binder solution.

The findings demonstrate that the fluidized bed agglomeration process markedly improved the reconstitution behavior of the coconut milk powder, particularly its solubility. The dissolution times of the agglomerated powders ranged between 5.15 and 5.56 min, notably shorter than that of the original powder (6.53 min). According to Freudig et al. [31], powder rehydration generally proceeds through three overlapping stages: wetting, dispersion, and solubilization. During the wetting stage, water is absorbed onto the surface of dry particles, allowing the powder to become wetted and to penetrate the surface of stationary water (Sharma et al. [32]). This step is succeeded by dispersion, in which the wetted particles fragment and become separated under gentle agitation [32]. During the final solubilization phase, the powder components are systematically dissolved into the surrounding aqueous medium [33]. As described by Ji et al. [15], the rate of wetting and dispersion is closely linked to structural features such as particle size, pore volume, and surface hydrophilicity, factors that collectively determine the ease of liquid penetration. In this context, the improved solubility observed in agglomerated samples can be attributed to the enlarged particle size and greater porosity generated during agglomeration, both of which enhance liquid access and internal diffusion. The lower tapped bulk density of the agglomerated powders further supports this explanation, confirming that the process yielded a more open and permeable powder structure conducive to rapid rehydration.

For a given binder formulation, no statistically significant difference was observed in the solubility of powders produced by the commercially manufactured and modified agglomeration systems. This outcome indicates that, although the two units differ in design and airflow configuration, and minor differences were noted in the bulk density of their products, similar reconstitution behavior can be attained when each system is operated under appropriately adjusted process conditions.

Agglomerates formed with distilled water as the binder dissolved more rapidly than those prepared using xanthan gum or xyloglucan solutions, despite their relatively higher bulk density. This behavior can be attributed to the development of a viscous or gel-like surface film when polysaccharide binders are employed, which likely impedes water penetration and delays the rehydration process. As highlighted by Ji et al. [15], the influence of binder composition on the wetting behavior of agglomerated powders is multifaceted; the molecular structure and polarity of the binder significantly affect how water interacts with and diffuses through the particle matrix during dissolution.

Although fluidized bed agglomeration noticeably enhanced the reconstitution properties of the coconut milk powder, the dissolution process of the agglomerated samples was still comparatively slow. This extended dissolution behavior can be ascribed to the high lipid content of the product, given that coconut milk powder produced from fresh coconut milk typically contains about 24% fat (U.S. Department of Agriculture [34]). The hydrophobic nature of lipids tends to hinder water uptake and slow down the overall dissolution rate. To address this limitation, the use of surface-active or hygroscopic additives may be beneficial. Fatimah et al. [27] demonstrated that higher concentrations of emulsifiers such as Tween 60 improved the emulsion stability of coconut milk and promoted faster wetting and dispersion of the resulting spray-dried powder.

The glass transition temperature is a critical thermal property influencing the structural and storage stability of powders and agglomerated materials. When the glass transition temperature is low, the amorphous components of the matrix tend to absorb more moisture and undergo a transition to a rubbery phase, resulting in stickiness, caking, and reduced flowability. These phenomena affect product handling, packaging efficiency, and shelf life. In contrast, maintaining a higher glass transition temperature keeps the material in a rigid, glassy state, thereby preserving its structural integrity throughout the storage and processing stages. Strategies to increase the thermal glass transition temperature generally involve decreasing residual moisture and employing high-molecular-weight compounds or carrier materials, such as polysaccharides and proteins, which can restrict molecular mobility and enhance powder stability [35].

**Figure 1:**
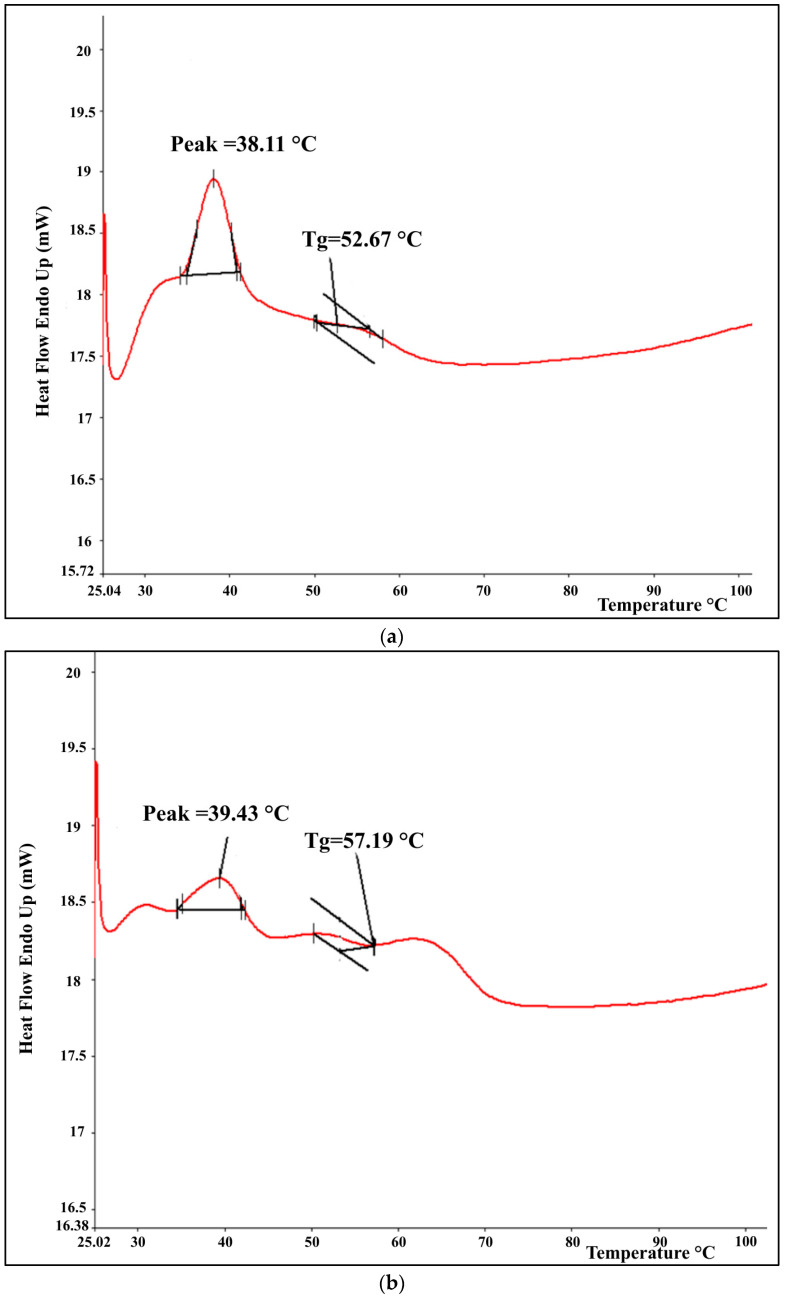

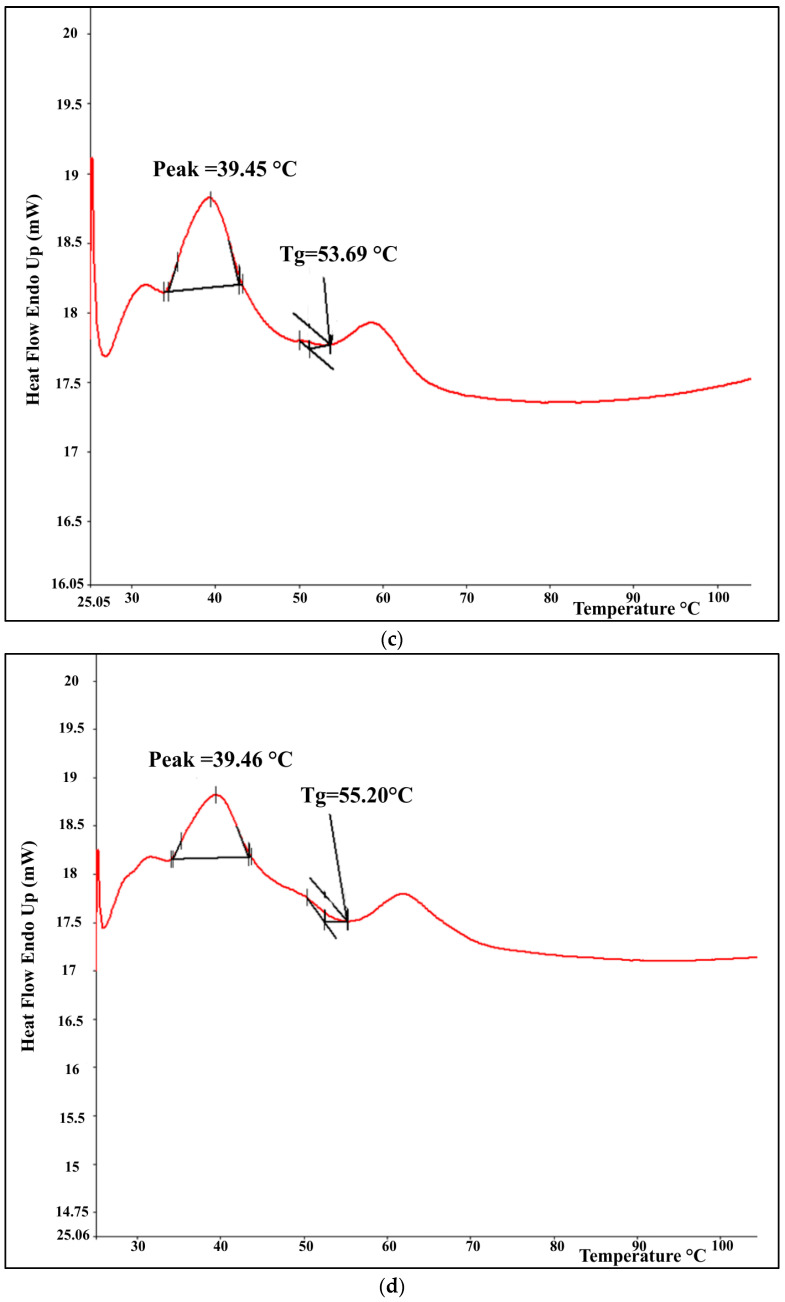
Representative DSC thermograms of coconut milk powder and selected agglomerated samples produced under different processing conditions: (**a**) non-agglomerated coconut milk powder, (**b**) agglomerate prepared with the commercially manufactured unit using distilled water as binder, (**c**) agglomerate produced with the modified unit using distilled water, and (**d**) agglomerate obtained with the modified unit using xanthan gum solution.

The glass transition temperature data for all samples are summarized in Table 3, while representative DSC thermograms are presented in Figure 1a–d. These include the profiles of the unagglomerated coconut milk powder, the water-based agglomerates produced using both fluidized bed systems, and the xanthan gum–bound agglomerate obtained from the modified unit. The overall thermal behavior among samples was generally comparable. The endothermic peak observed at approximately 38–40 °C is attributed primarily to the melting of lipid constituents in the coconut milk matrix. Coconut oil, the predominant fat in coconut milk, typically melts between 24 °C and 26 °C due to its high content of medium-chain triglycerides, particularly lauric acid [36,37]. However, in powder form, these lipids are encapsulated within a complex matrix of carbohydrates and proteins, which can cause a shift or broadening of the melting peak. This behavior is influenced by interactions with carrier substances such as glucose syrup and sodium caseinate, as indicated on the product label. The addition of hydrocolloid binders, including xanthan gum and xyloglucan, during agglomeration may also influence this thermal response by altering the physical network of the powder. Despite such matrix effects, the transition observed near 38–40 °C corresponds more closely to lipid melting rather than to the glass transition, which is typically manifested as a change in baseline slope rather than a discrete endothermic peak, as depicted in Figure 1.

All agglomerated powders exhibited notably higher glass transition temperatures than the unprocessed coconut milk powder. This enhancement can be attributed to structural modifications that occur during the fluidized bed agglomeration process and is further influenced by the incorporation of polysaccharide binders, including xanthan gum and xyloglucan. These macromolecules can establish hydrogen bonds and other intermolecular interactions with the components of the powder matrix, leading to enhanced rigidity and reduced molecular mobility, factors that collectively raise the glass transition temperature. Reported glass transition temperature values for xanthan gum and xyloglucan are approximately 105–116 °C [38,39] and about 270 °C [40], respectively. The inherently high transition temperatures of these biopolymers therefore help explain the increase in the overall glass transition temperatures of the agglomerated samples when they are incorporated as binders.

Agglomerates produced with the modified hybrid-dryer system showed lower glass transition temperatures than those obtained from the commercially manufactured agglomerator, regardless of the binder used. This difference is likely related to variations in microstructural development caused by distinct operating conditions, such as binder feed rate, total liquid addition, nozzle design, and airflow intensity, between the two systems. Because these factors interact in a complex and interdependent manner, their collective influence on agglomerate morphology and thermal behavior was interpreted as an overall process effect rather than evaluated individually. Furthermore, the incorporation of xanthan gum and xyloglucan binders tended to produce powders with slightly higher glass transition temperatures values than those prepared with distilled water, a trend most evident for samples generated using the commercially manufactured agglomerator.

Overall, the findings from this study demonstrate that both the custom-modified and commercially manufactured fluidized bed agglomerators were effective in enhancing the physical quality of coconut milk powder. Although the two systems yielded comparable results for most measured properties, the commercially manufactured unit produced powders with a distinctly higher glass transition temperature, indicating potentially greater resistance to structural changes during storage. The inclusion of xanthan gum and xyloglucan as binders marginally extended the dissolution time relative to water-based formulations; however, their positive impact on increasing glass transition temperature suggests a trade-off that could favor improved product stability over prolonged storage.

## 4. Conclusions

The present work confirms that fluidized bed agglomeration is an effective method for improving the physical and functional characteristics of coconut milk powder. All samples, including the untreated powder and agglomerated products, exhibited moisture contents ranging between 2.1% and 2.6% w.b., which are sufficiently low to ensure stability during ambient storage. Agglomeration markedly decreased tapped bulk density, evidencing the formation of expanded, highly porous granules. Among the binders investigated, xanthan gum and xyloglucan produced powders with the lowest bulk density, whereas distilled water resulted in agglomerates with the fastest dissolution rate. Although solubility did not differ significantly between the two agglomeration systems when the same binder was used, overall reconstitution performance was superior to that of the original powder. DSC revealed an endothermic transition at approximately 38–40 °C, attributed to lipid melting, while the glass transition temperatures (51.7–61.7 °C) were consistently higher for all agglomerated powders than for the non-agglomerated sample. Slightly lower glass transition temperature values were obtained from the modified hybrid-dryer system compared with the commercially manufactured unit, regardless of binder type. Overall, these results demonstrate that both systems can successfully enhance powder quality, with the commercially manufactured unit yielding marginally higher thermal stability. Future studies should extend this approach to a wider range of plant-based ingredients to further assess its versatility and scalability. Detailed investigations of agglomerate morphology, particle size distribution, and growth behavior, together with systematic optimization of key process parameters, would provide deeper insight into the agglomeration mechanism and support process scale-up.

## Figures and Tables

**Table 1:** Operating parameters applied in the formation of agglomerates from coconut milk powder using both the commercially manufactured and the modified agglomerators.

Condition No.	Agglomerator Type	Binder Type	Rate of Binder Addition (mL/min)	Binder Volume Delivered Through Spraying (mL)	Drying Duration (min)
1	Commercially manufactured unit	Distilled water	4.0	92	50
2		Xanthan gum solution	4.9	64	60
3		Xyloglucan solution	4.0	76	50
4	Modified unit	Distilled water	5.6	50	60
5		Xanthan gum solution	7.6	68	40
6		Xyloglucan solution	5.0	70	45

**Table 2:** Key specifications and operational parameters of the commercially manufactured and the modified agglomerators used in this study.

Parameter	Commercially Manufactured Agglomerator	Modified Agglomerator
Sample mass of coconut milk powder for each experiment (g)	100	100
Type of binder atomizing nozzle	Two-fluid nozzle	Two-fluid nozzle
Nozzle orifice diameter (mm)	0.8	0.4
Pressure of air supplied to nozzle (MPa)	0.2	0.2
Nozzle position above distributor plate (cm)	12	7
Distributor plate diameter (cm)	7.5	30
Drying air temperature (°C)	50	50
Drying air velocity range (m/s)	0.5–1.8	1.5–3.0

Remark: Both systems operated under batch conditions with identical binder types and drying temperatures. Differences in nozzle geometry, plate dimensions, and airflow velocity reflect the specific design features of each agglomerator.

**Table 3:** Physicochemical characteristics of the raw and agglomerated coconut milk powders produced with different binders and agglomeration systems.

No.	Agglomerator Type	Sample Description	Moisture Content (% w.b.)	Tapped Bulk Density (g/mL)	Solubility (min)	Differential Scanning Calorimetry		
						Onset Temperature (°C)	Glass Transition Temperature (°C)	End Temperature (°C)
1	N/A	Coconut milk powder (unagglomerated)	2.2 ^b^ ± 0.01	0.577 ^e^ ± 0.01	6.53 ^c^ ± 0.05	50.23 ^a^ ± 0.04	51.67 ^a^ ± 1.41	56.19 ^c^ ± 0.40
2	Commercially manufactured unit	Agglomerate (binder: distilled water)	2.2 ^b^ ± 0.01	0.480 ^d^ ± 0.01	5.29 ^a^ ± 0.21	53.97 ^c^ ± 1.13	57.02 ^c^ ± 0.24	57.09 ^c^ ± 0.23
3		Agglomerate (binder: xanthan gum solution)	2.6 ^d^ ± 0.04	0.465 ^c^ ± 0.00	5.53 ^b^ ± 0.04	57.14 ^d^ ± 1.13	59.66 ^d^ ± 0.35	60.08 ^d^± 0.12
4		Agglomerate (binder: xyloglucan solution)	2.4 ^c^ ± 0.01	0.448 ^b^ ± 0.01	5.56 ^b^ ± 0.03	56.34 ^d^ ± 0.47	61.67 ^e^ ± 0.12	64.20 ^e^ ± 0.23
5	Modified unit	Agglomerate (binder: distilled water)	2.1 ^a^ ± 0.01	0.462 ^c^ ± 0.01	5.15 ^a^ ± 0.05	50.86 ^ab^ ± 0.49	53.35 ^b^ ± 0.49	53.54 ^a^ ± 0.31
6		Agglomerate (binder: xanthan gum solution)	2.5 ^d^ ± 0.01	0.420 ^a^ ± 0.01	5.49 ^b^ ± 0.09	52.15 ^b^ ± 0.38	54.86 ^b^ ± 0.48	54.91 ^b^ ± 0.53
7		Agglomerate (binder: xyloglucan solution)	2.3 ^c^ ± 0.04	0.432 ^a^ ± 0.01	5.52 ^b^ ± 0.08	51.56 ^ab^ ± 0.75	54.76 ^b^ ± 0.59	54.95 ^b^ ± 0.76

Remark: N/A = not applicable. Values represent mean ± standard deviation (*n* = 3). Different lowercase superscripts within the same column denote statistically significant differences among samples (*p* ≤ 0.05).

## Data Availability

The data supporting the findings of this study are available from the corresponding author upon reasonable request.
